# Clinical Significance of Corneal Striae in Thyroid Associated Orbitopathy

**DOI:** 10.3390/jcm12062284

**Published:** 2023-03-15

**Authors:** Xulin Liao, Fatema Mohamed Ali Abdulla Aljufairi, Kenneth Ka Hei Lai, Karen Kar Wun Chan, Ruofan Jia, Wanxue Chen, Zhichao Hu, Yingying Wei, Winnie Chiu Wing Chu, Clement Chee Yung Tham, Chi Pui Pang, Kelvin Kam Lung Chong

**Affiliations:** 1 Department of Ophthalmology and Visual Sciences, The Chinese University of Hong Kong, Hong Kong SAR, China; 2 Department of Ophthalmology, Salmaniya Medical Complex, Government Hospitals, Manama 435, Bahrain; 3 Department of Ophthalmology, Tung Wah Eastern Hospital, Hong Kong SAR, China; 4 Department of Ophthalmology and Visual Sciences, Prince of Wales Hospital, Hong Kong SAR, China; 5 Department of Statistics, The Chinese University of Hong Kong, Hong Kong SAR, China; 6 Department of Imaging and Interventional Radiology, Faculty of Medicine, The Prince of Wales Hospital, The Chinese University of Hong Kong, Hong Kong SAR, China

**Keywords:** thyroid associated orbitopathy, corneal striae, lagophthalmos, extraocular muscle, levator palpebrae superioris–superior rectus complex

## Abstract

Purpose: To elucidate the clinical implications of corneal striae (CS) in thyroid associated orbitopathy (TAO) patients. Methods: In this cross-sectional study, the presence of CS was confirmed after topical fluorescein staining on a slit lamp for consecutive treatment-naive TAO patients. Orbital parameters, including margin reflex distances, lagophthalmos, exophthalmos, intraocular pressure and radiological measurements, were compared between eyes with and without CS. The largest cross-sectional areas of each rectus muscle were measured by segmenting the T1-weighted (T1W) magnetic resonance images (MRI). The logistic regression analyses were used to evaluate the associations between CS and orbital parameters and rectus muscle measurements. Results: Fifty-three consecutive TAO patients (presenting age 46.47 ± 14.73 years, clinical activity score 1.77 ± 1.25) who had unilateral CS were enrolled. In univariate analysis, both the degree of lagophthalmos and the area of the levator palpebrae superioris–superior rectus complex (LPS/SR) on T1W MRI were significantly larger in CS eyes compared to eyes without CS (*p* < 0.05). Multivariate analyses showed that CS in TAO patients were significantly associated with the degree of lagophthalmos (OR = 1.75, 95% CI: 1.18–2.61, *p* < 0.05) and LPS/SR area (OR = 19.27, 95% CI: 1.43–259.32, *p* < 0.05) but not with the other parameters. CS could predict LPS/SR enlargement and larger lagophthalmos in TAO (*p* < 0.05). The largest cross-sectional areas of LPS/SR and inferior rectus were positively correlated with clinical activity scores (*p* < 0.05). Conclusions: The presence of CS in TAO eye is significantly associated with LPS/SR enlargement and worse lagophthalmos. CS might be evaluated further as a potential ocular surface biomarker to identify upper lid and LPS/SR involvement in TAO.

## 1. Introduction

Thyroid associated orbitopathy (TAO), also known as thyroid eye disease and Grave’s orbitopathy [1], is an autoimmune disorder primarily involving orbital soft tissues. Around 80% of cases are associated with hyperthyroidism [2], and the remaining 20% are associated with either hypothyroidism, Hashimoto’s disease or even euthyroidism. TAO is characterized by inflammatory expansion and infiltration of retro-ocular tissues within the orbits, the expansion of adipose tissues within or around the eye muscles and the increase in extraocular muscles and orbital fat chondroitin sulphate and hyaluronan deposition due to excess production of glycosaminoglycan [3]. TAO is often a biphasic disease, which typically begins with an active inflammatory stage that lasts for 12–24 months, followed by a chronic stable fibrotic stage [4,5]. 

The diagnosis of TAO is essentially based on clinical examinations. The severity of the disease may range from a mild form, which manifests in eyelid and soft tissue involvement, to a very severe form, including sight-threatening globe subluxation, exposure keratopathy [6] and dysthyroid optic neuropathy [7]. These complications often occur due to the delay in diagnosis and treatment. Clinical activity score (CAS) is widely used to grade the inflammatory activity of TAO; a score of 3 or more is considered as active disease. However, patients with low CAS who progressed and developed severe TAO were increasingly reported [8]. Orbital imaging techniques, especially magnetic resonance imaging (MRI), are found useful in quantifying disease activity and severity [9]. However, these modalities are costly, time consuming, and high-quality images are not easily available. 

**Figure 1:**
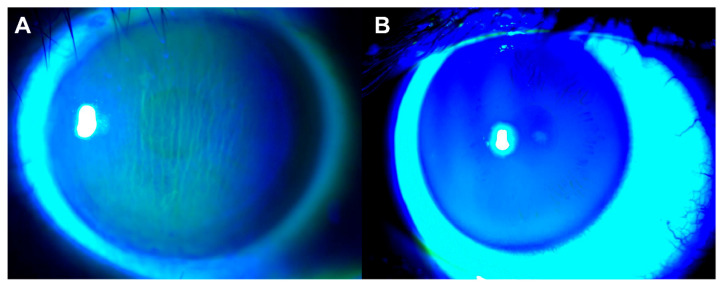
Corneal striae were determined during slit-lamp examination after sodium fluorescence staining. (**A**) Corneal striae in TAO. (**B**) Normal cornea in TAO.

Corneal striae were first reported in an oriental female from a UK group [10] and later by our group in Chinese patients [11]. They are vertical wrinkle-like streaks on the corneal epithelial layer. They are best observed after topical fluorescence staining under cobalt blue light using slit-lamp microscopy (Figure 1), which is simple, non-invasive and widely available in ophthalmology clinics. The clinical implications of corneal striae in TAO remain understudied. Herein, we compare the eyes of the same individual TAO patients who presented with unilateral corneal striae, comparing their orbital and radiological parameters.

## 2. Methods

This is a cross-sectional study. Only treatment-naive TAO patients with unilateral corneal striae were analyzed. Patients were consecutively recruited from a TAO cohort of over 1300 Chinese patients in Hong Kong established from 2012 to 2022. The patients were recruited from the Chinese University of Hong Kong Eye Centre, the Eye Centre at the Chinese University of Hong Kong Medical Centre and the Eye Centre at the Prince of Wales Hospital. This study adhered to the tenets of the Declaration of Helsinki and the Ethics approvals (KC/KE-10-0218/ER-3, NTEC Ref. 2010.594) obtained from the Chinese University of Hong Kong. We included patients with clinical diagnoses of TAO presented to a single oculoplastic surgeon [9,12]. We excluded patients with incomplete clinical or radiological data. 

### 2.1. Orbital Examination

The orbital examination included margin reflex distance to the upper eyelid (MRD1), margin reflex distance to the lower eyelid (MRD2), lagophthalmos and clinical activity score (CAS) [13]. The Hertel exophthalmometer was used to measure the degree of exophthalmos [14]. The Goldmann Applanation Tonometer was used to measure the intraocular pressure (IOP) in primary and upgaze [15].

### 2.2. Magnetic Resonance Imaging

Orbital MRI was arranged for all subjects recruited for this study during their first visit shortly after the initial orbital and slit-lamp examination was performed. MRI was performed using either the 3.0 T Siemens scanner (MAGNETOM Prisma, Siemens, Erlangen, Germany) or the 3.0 T Philips scanner (Achieva TX; Philips Healthcare, Best, The Netherlands). In the 3.0 T Siemens scanner (MAGNETOM Prisma, Siemens, Erlangen, Germany), a 64-channel head/neck coil was used. Coronal T1WI pre-contrast T1-weighted imaging was conducted using the turbo spin echo (TSE) technique at the coronal plane: repetition time (TR)/echo time (TE) = 585/16 ms; acceleration factor for phase encoding (Accel. factor PE) = 3; voxel size = 0.2 × 0.2 mm; matrix = 384 × 307; slice thickness = 3.0 mm; slice number = 26; flip angle = 130°; number of averages = 3. Meanwhile, in the 3.0 T Philips scanner (Achieva TX; Philips Healthcare, Best, the Netherlands), 16-channel Philips neurovascular phased-array coils were used. Coronal T1WI pre-contrast T1-weighted imaging was conducted using the turbo spin echo (TSE) technique at the coronal plane: repetition time (TR)/echo time (TE) = 642/13 ms; TSE factor = 4; slice thickness/gap = 3.0 mm/0.3 mm; slice number = 26; voxel size = 0.3 × 0.3 mm; matrix = 300 × 239; flip angle = 90°; NSA = 1. The type of MRI machine used was largely based on the availability at the MRI center. The patients were instructed to close their eyes and remain motionless during scanning, after stabilizing their heads in a supine position.

**Figure 2:**
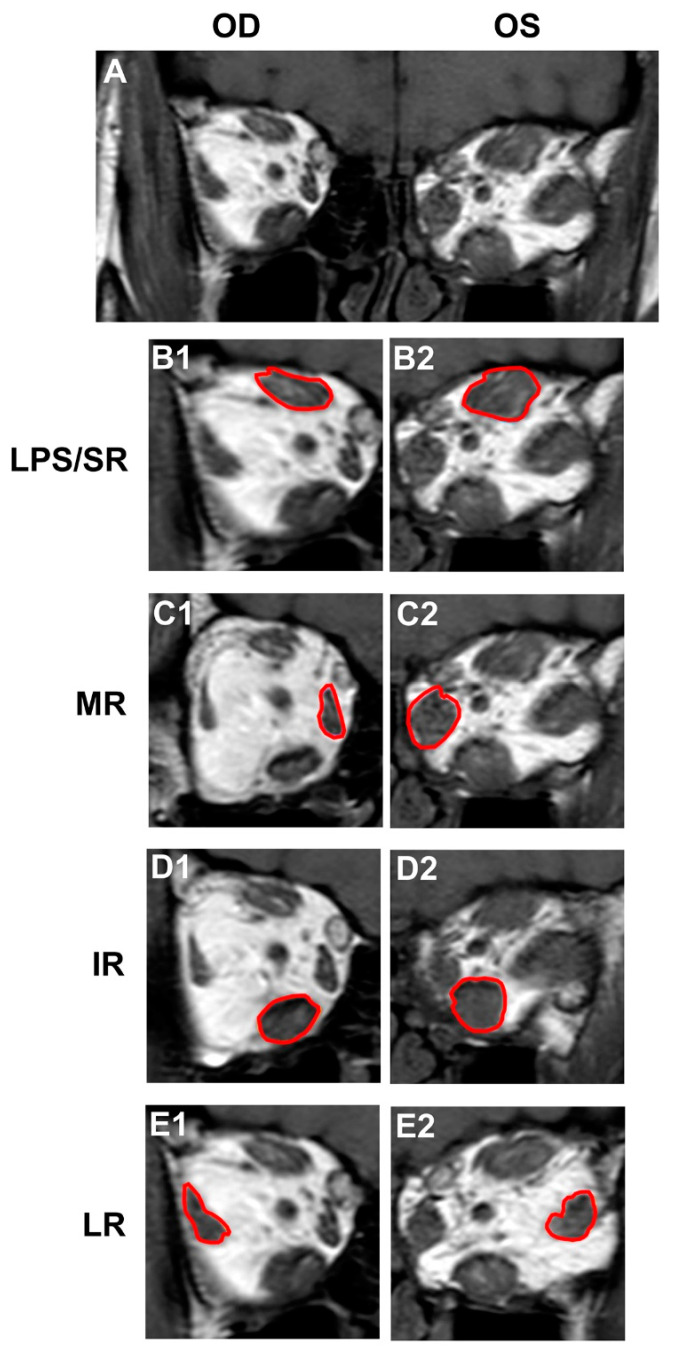
The measurement of the largest cross-sectional area of the extraocular muscle on T1-weighted MR images. (**A**). Representative T1W orbital MR image. (**B1**,**B2**). Segmentation of the largest LPS/SR image. (**C1**,**C2**). Segmentation of the largest medial rectus (MR) image. (**D1**,**D2**). Segmentation of the largest lateral rectus (LR) image. (**E1**,**E2**). Segmentation of the largest inferior rectus (IR) image. Red line represents segmentation of extraocular muscles. LPS/SR, levator palpebrae superioris–superior rectus complex.

T1-weighted (T1W) coronal images were used to measure the cross-sectional area of individual extraocular muscles, which included medial rectus (MR), inferior rectus (IR), lateral rectus (LR) and levator palpebrae superioris–superior rectus complex (LPS/SR). This has been regarded as the best sequence to delineate the orbital muscle anatomy [16]. The coronal section with the largest cross-sectional area for each muscle belly was chosen for measurement. The region of interest (ROI) was manually traced around the surface of each extraocular muscle by a single oculoplastic surgeon who was blinded to the clinical examination findings using a dedicated workstation (Syngo. Via, Siemens, Erlangan, Germany). Each muscle area was measured 3 times, and the mean value was taken (Figure 2).

### 2.3. Statistical Analyses

The data analyses were performed using the IBM SPSS 23.0 (IBM SPSS Inc., Armonk, NY, USA) and R (The R Project for Statistical Computing, version 4.2.1). Graphs were generated using GraphPad Prism 8.0.1 (GraphPad Software, La Jolla, CA, USA). Continuous variables were described using means ± standard deviations. To compare the difference between two groups, the Chi-square test was used for categorical variables, and the paired t-test was used for continuous variables. The univariate and multivariate logistic regression analyses were used to detect the association between corneal striae and measurement parameters in TAO eyes. The generalized estimating equation was used to correct inter-eye correlation. The ROC curve was used to analyze the effect of IOP, orbital parameters and the area of EOM to differentiate between the eyes with or without corneal striae from each TAO patient. We also applied linear regression analysis to investigate the relationship between the CAS and the largest cross-sectional area of EOM in TAO patients. Results were considered statistically significant if the *p*-value < 0.05.

## 3. Results

Out of our cohort of 1300 patients with TAO, 53 (4.1%) had unilateral corneal striae, and 338 (26.0%) had corneal striae present in at least one eye. In our study, we analyzed a total of 53 TAO patients (106 eyes) with unilateral corneal striae. The mean age at evaluation was 48.83 ± 14.46 years, the age at TAO onset was 46.47 ± 14.73 years, and the clinical activity score presented was 1.77 ± 1.25. Among them, 32.1% (17/53) were smokers, and 92.5% (49/53) had Graves’ disease. The demographic characteristics of 53 TAO patients with unilateral corneal striae are summarized in Table 1. It is noted that TAO-related corneal striae are vertically oriented.

We found that eyes with corneal striae always had more lagophthalmos (0.83 ± 1.10 mm vs. 0.42 ± 0.69 mm, *p* = 0.022) and larger LPS/SR cross-sectional area (0.52 ± 0.21 cm^2^ vs. 0.42 ± 0.21 cm^2^, *p* = 0.003) than eyes without corneal striae from the same individual (Table 2).

Using univariate logistic regression, the odds ratio of lagophthalmos was found to be 1.69, and the 95% CI was 1.17 to 2.45, with *p* = 0.005. The odds ratio of the cross-sectional area of the LPS/SR was 12.70, and the 95% CI was 1.15 to 140.47, *p* = 0.038 (Table 3).

After multivariate logistic regression adjusting for age, sex, Graves’ disease status and smoking habits, we found that the odds ratio of lagophthalmos was 1.75, and the 95% CI was 1.18 to 2.61, *p* = 0.006. This was translated into a 75% increase in the risk of developing corneal striae for every 1 mm increase in the degree of lagophthalmos. This also means that among TAO eyes, those with corneal striae had worse lagophthalmos than those without corneal striae. The odds ratio of the cross-sectional area of the LPS/SR was 19.27, and the 95% CI was 1.43 to 259.32, *p* = 0.026. This indicated that in eyes with TAO, every 1 mm^2^ enlargement of the LPS/RS complex was associated with an 18-fold increase in the risk of presenting corneal striae. This also means that in eyes with TAO, those with corneal striae had larger LPS/RS cross-sectional areas than those without corneal striae (Table 3). However, the IOP, MRD, exophthalmos and other EOM enlargements, including the medial rectus, lateral rectus and inferior rectus, did not show significant differences between eyes with and without corneal striae.

**Figure 3:**
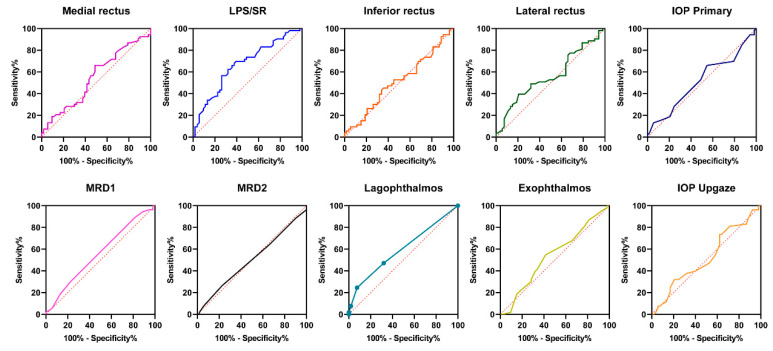
Receiver operating characteristic (ROC) curve analysis showing the area under the curve (AUC) of the diagnostic confidence for different parameters. IOP, intraocular pressure; MRD1, margin reflex distance to the upper eyelid; MRD2, margin reflex distance to the lower eyelid; LPS/SR, levator palpebrae superioris–superior rectus complex.

The results of the ROC curve analysis showed that the corneal striae could be used as a potential predictive marker for LPS/SR enlargement (area under the ROC curve (AUC) = 0.666, 95% CI = 0.562–0.769, *p* = 0.001) and lagophthalmos (AUC = 0.598, 95% CI = 0.503–0.692, *p* = 0.004) in TAO patients (Figure 3 and Table 4).

**Figure 4:**
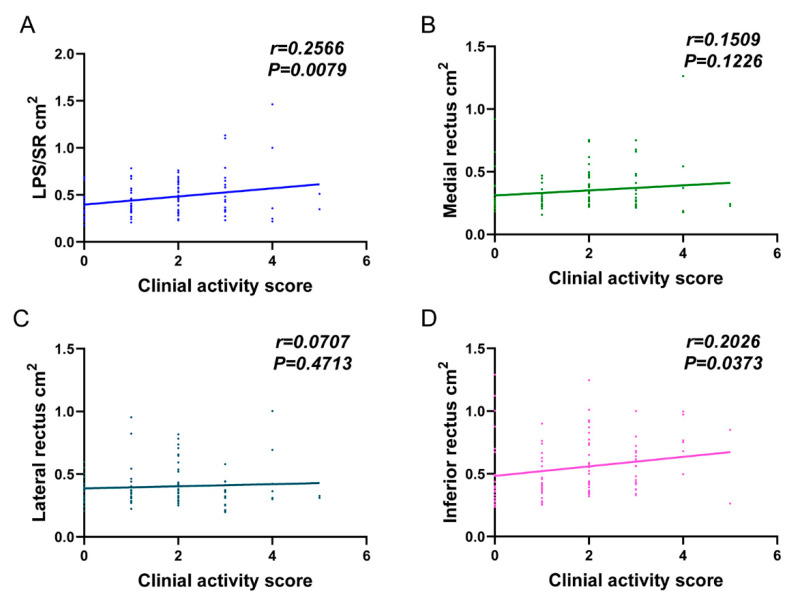
Linear regression analysis of the changes in clinical activity scores and the largest cross-sectional area of extraocular muscles. (**A**) LPS/SR; (**B**) Medial rectus; (**C**) Lateral rectus; (**D**) Inferior rectus) in 53 TAO patients. IOP, intraocular pressure; MRD1, margin reflex distance to the upper eyelid; MRD2, margin reflex distance to the lower eyelid; LPS/SR, levator palpebrae superioris–superior rectus complex.

Linear regression analysis showed that the largest cross-sectional areas of LPS/SR (r = 0.2566, *p* = 0.0079) and inferior rectus (r = 0.2026, *p* = 0.0373) were positively correlated with the clinical activity score in TAO patients (Figure 4).

## 4. Discussion

Published data on the clinical significance of corneal striae in TAO patients are limited. We noticed reports of corneal striae in two TAO patients [9,10]. Corneal striae were also reported in different ophthalmic diseases, such as primary congenital glaucoma [17], keratoconus [18], Fuchs’ endothelial corneal dystrophy [19] and post-LASIK corneal striae [20]. However, corneal striae in our study were only presented vertically, different from the post-LASIK corneal striae with lines resembling fine lattice [20] or Haab’s striae with thickened parallel cord-like lines [21]. Our study also showed that the TAO eye with corneal striae was significantly associated with worse lagophthalmos and larger LPS/SR.

Lagophthalmos is the incomplete or defective closure of the eyelids. It is a common manifestation of TAO due to eyelid retraction and/or exophthalmos. On the other hand, it can also present in other eye diseases, such as facial nerve paralysis [22], after trauma or surgery [23]. TAO patients with severe lagophthalmos are prone to increased tear aqueous evaporation, and thus, evaporative dry eye symptoms.

TAO is an autoimmune-driven orbital disease, which results in the infiltration and enlargement of extraocular muscles, orbital fat, eyelid and lacrimal glands. MRI is now increasingly used in TAO to provide an objective evaluation of the inflammatory involvement and/or enlargement of extraocular muscles, lacrimal glands and orbital fat [24]. In this study, we found that for the same TAO patients, the eyes with corneal striae always have larger LPS/SR. An increase in LPS/SR area by 1 mm^2^ increases the risk of corneal striae by around 18-fold. Our findings indicate that corneal epithelial changes, readily captured by slit-lamp biomicroscopes, may predict orbital radiological abnormalities, in this case, an enlarged LPS/SR complex. We speculate that the enlargement of the LPS/SR results in undue pressure on the cornea, leading to the development of corneal striae.

Previous studies on corneal striae were associated with abnormal intraocular pressure, especially with low IOP being reported [25,26]. In this study, the IOP (including in primary and upgaze) was not associated with corneal striae. The physiology and biomechanics of corneal striae in TAO need further investigation.

There are many disease markers for TAO, such as serum thyroid-stimulating immunoglobulin (TSI) [27] and orbital images, including MRI [28]. However, in our study, we found that corneal striae, which can be easily observed under a slit lamp, are an indicator of underlying LPS/SR muscle enlargement and upper eyelid involvement. This is useful for identifying patients with a higher likelihood of severe TAO. The presence of corneal striae will help triage patients for earlier assessment by oculoplastic surgeons and/or arrange orbital MR imaging examination for better assessment of disease status.

Our study revealed a positive correlation between the largest cross-sectional area of LPS/SR and the clinical activity score in patients with TAO. We also observed that the enlargement of LPS/SR was related to corneal striae, suggesting a potential positive association between CS and the clinical activity score in TAO patients. Further studies are needed to confirm whether corneal striae alone or in combination may serve as a potential disease marker of TAO severity and activity, as well as the biomechanical basis of corneal striae. Additionally, further research is needed to determine whether CS will disappear after treatment with decompression surgery, eyelid surgery or lubricating eye drops. There are logistic merits to evaluating corneal striae under a slit lamp; namely, it is a simple, non-invasive and readily available technique in eye clinics.

In conclusion, we comprehensively studied a “new” clinical feature in TAO patients, the corneal striae. Our results showed that corneal striae are vertical and significantly associated with worse lagophthalmos and larger LPS/SR cross-sectional area. Corneal striae might be evaluated as a non-invasive biomarker to determine TAO severity and activity.

## Figures and Tables

**Table 1:** Demographic characteristics of 53 TAO patients with unilateral corneal striae.

Parameters	Patients with TAO
Number of patients	53
Number of eyes	106
Number of CS eyes (N%)	53 (50%)
Age (years)	48.83 ± 14.46
Female:Male	36:17
Onset age of TAO (years)	46.47 ± 14.73
Clinical activity score	1.77 ± 1.25
Graves’ disease (N%)	49 (92.5%)
Smoker (N%)	17 (32.1%)

TAO, thyroid associated orbitopathy; CS, corneal striae.

**Table 2:** Comparison of measurement results between TAO eyes with and without corneal striae in 53 TAO patients.

	TAO with CS	TAO without CS	*p*-Value
Number of eyes	53	53	
BCVA (Log MAR)	0.13 ± 0.20	0.08 ± 0.18	0.143
IOP Primary (mmHg)	17.15 ± 3.77	16.94 ± 3.34	0.765
IOP Upgaze (mmHg)	25.53 ± 6.37	24.91 ± 6.56	0.704
**Orbital parameters**			
MRD1 (mm)	5.60 ± 1.98	5.15 ± 2.06	0.277
MRD2 (mm)	5.15 ± 1.22	5.11 ± 1.12	0.974
Lagophthalmos (mm)	0.83 ± 1.10	0.42 ± 0.69	0.022
Exophthalmos (mm)	18.84 ± 2.43	18.52 ± 2.77	0.484
**Extraocular muscles**			
LPS/SR (cm^2^)	0.52 ± 0.21	0.42 ± 0.21	0.003
Medial rectus (cm^2^)	0.36 ± 0.20	0.33 ± 0.13	0.401
Lateral rectus (cm^2^)	0.39 ± 0.15	0.41 ± 0.15	0.270
Inferior rectus (cm^2^)	0.56 ± 0.25	0.54 ± 0.23	0.845

TAO, thyroid associated orbitopathy; CS, corneal striae; BCVA, best corrected visual acuity; IOP, intraocular pressure; MRD1, margin reflex distance to the upper eyelid; MRD2, margin reflex distance to the lower eyelid; LPS/SR, levator palpebrae superioris–superior rectus complex.

**Table 3:** Association of corneal striae and measurement parameters in TAO eyes (N = 106).

	Univariate Model	Multivariate Model *
	OR (95% CI) *p*-Value	OR (95% CI) *p*-Value
IOP Primary (mmHg)	1.02 (0.95, 1.09) 0.645	1.02 (0.94, 1.10) 0.646
IOP Upgaze (mmHg)	1.02 (0.98, 1.05) 0.358	1.02 (0.98, 1.05) 0.360
**Orbital parameters**		
MRD1 (mm)	1.12 (0.98, 1.28) 0.103	1.13 (0.98, 1.32) 0.101
MRD2 (mm)	1.03 (0.85, 1.24) 0.770	1.03 (0.85, 1.25) 0.770
Lagophthalmos (mm)	1.69 (1.17, 2.45) 0.005	1.75 (1.18, 2.61) 0.006
Exophthalmos (mm)	1.05 (0.98, 1.12) 0.172	1.06 (0.98, 1.14) 0.171
**Extraocular muscles**		
LPS/SR (cm^2^)	12.70 (1.15, 140.47) 0.038	19.27 (1.43, 259.32) 0.026
Medial rectus (cm^2^)	3.61 (1.00, 12.95) 0.049	5.18 (0.93, 28.94) 0.061
Lateral rectus (cm^2^)	0.38 (0.08, 1.87) 0.235	0.31 (0.04, 2.16) 0.238
Inferior rectus (cm^2^)	1.43 (0.53, 3.85) 0.474	1.65 (0.42, 6.47) 0.476

TAO, thyroid associated orbitopathy; IOP, intraocular pressure; MRD1, margin reflex distance to the upper eyelid; MRD2, margin reflex distance to the lower eyelid; LPS/SR, levator palpebrae superioris–superior rectus complex. * Multivariate model adjusted for age, sex, Graves’ disease, smoker.

**Table 4:** Receiver operating characteristic (ROC) curve analysis of TAO eyes (N = 106).

	AUC	95% CI	*p*-Value
IOP Primary (mmHg)	0.517	0.406–0.628	0.678
IOP Upgaze (mmHg)	0.479	0.367–0.590	0.390
**Orbital parameters**			
MRD1 (mm)	0.560	0.452–0.669	0.089
MRD2 (mm)	0.498	0.392–0.604	0.752
Lagophthalmos (mm)	0.598	0.503–0.692	0.004
Exophthalmos (mm)	0.539	0.429–0.650	0.074
**Extraocular muscles**			
LPS/SR (cm^2^)	0.666	0.562–0.769	0.001
Medial rectus (cm^2^)	0.547	0.436–0.658	0.179
Lateral rectus (cm^2^)	0.562	0.451–0.673	0.305
Inferior rectus (cm^2^)	0.511	0.400–0.622	0.227

AUC, area under the ROC curve; IOP, intraocular pressure; MRD1, margin reflex distance to the upper eyelid; MRD2, margin reflex distance to the lower eyelid; LPS/SR, levator palpebrae superioris–superior rectus complex.

## Data Availability

Not applicable.
